# MR-Guided Focused Ultrasound for Refractory Epilepsy: Where Are We Now?

**DOI:** 10.3390/jcm12227070

**Published:** 2023-11-13

**Authors:** Angelo Labate, Salvatore Bertino, Rosa Morabito, Chiara Smorto, Annalisa Militi, Simona Cammaroto, Carmelo Anfuso, Francesco Tomaiuolo, Paolo Tonin, Silvia Marino, Antonio Cerasa, Angelo Quartarone

**Affiliations:** 1 Neurophysiopathology and Movement Disorders Unit, BIOMORF Department, University of Messina, 98124 Messina, Italy; angelo.labate@unime.it; 2 Department of Clinical and Experimental Medicine, University of Messina, 98122 Messina, Italy; bertinosalvatore404@gmail.com (S.B.); francesco.tomaiuolo@gmail.com (F.T.); 3 IRCCS Centro Neurolesi “Bonino Pulejo”, 98124 Messina, Italy; rosa.morabito@irccsme.it (R.M.); chiara.smorto@irccsme.it (C.S.); militiannalisa@gmail.com (A.M.); simona.cammaroto@irccsme.it (S.C.); carmelo.anfuso@irccsme.it (C.A.); silvia.marino@irccsme.it (S.M.); aquartar65@gmail.com (A.Q.); 4 S.Anna Institute, 88900 Crotone, Italy; patonin18@gmail.com; 5 Institute for Biomedical Research and Innovation (IRIB), National Research Council of Italy, 98164 Messina, Italy; 6 Pharmacotechnology Documentation and Transfer Unit, Preclinical and Translational Pharmacology, Department of Pharmacy, Health Science and Nutrition, University of Calabria, 87036 Rende, Italy

**Keywords:** MRgFUS, intractable epilepsy, ablation, low-frequency modulation

## Abstract

Epilepsy is one of the most common neurological diseases in both adults and children. Despite improvements in medical care, 20 to 30% of patients are still resistant to the best medical treatment. The quality of life, neurologic morbidity, and even mortality of patients are significantly impacted by medically intractable epilepsy. Nowadays, conservative therapeutic approaches consist of increasing medication dosage, changing to a different anti-seizure drug as monotherapy, and combining different antiseizure drugs using an add-on strategy. However, such measures may not be sufficient to efficiently control seizure recurrence. Resective surgery, ablative procedures and non-resective neuromodulatory (deep-brain stimulation, vagus nerve stimulation) treatments are the available treatments for these kinds of patients. However, invasive procedures may involve lengthy inpatient stays for the patients, risks of long-term neurological impairment, general anesthesia, and other possible surgery-related complications (i.e., hemorrhage or infection). In the last few years, MR-guided focused ultrasound (MRgFUS) has been proposed as an emerging treatment for neurological diseases because of technological advancements and the goal of minimally invasive neurosurgery. By outlining the current knowledge obtained from both preclinical and clinical studies and discussing the technical opportunities of this therapy for particular epileptic phenotypes, in this perspective review, we explore the various mechanisms and potential applications (thermoablation, blood-brain barrier opening for drug delivery, neuromodulation) of high- and low-intensity ultrasound, highlighting possible novel strategies to treat drug-resistant epileptic patients who are not eligible or do not accept currently established surgical approaches. Taken together, the available studies support a possible role for lesional treatment over the anterior thalamus with high-intensity ultrasound and neuromodulation of the hippocampus via low-intensity ultrasound in refractory epilepsy. However, more studies, likely conceiving epilepsy as a network disorder and bridging together different scales and modalities, are required to make ultrasound delivery strategies meaningful, effective, and safe.

## 1. Treatment of Epilepsy

Epilepsy is one of the most common neurological diseases in both adults and children. Despite advances in treatment, almost 20 to 30% of patients are resistant to the medications [1]. In the epilepsy field, drug resistance is defined as the failure of at least two anti-seizure drugs administered in a proper dosage, either alone or in combination [2,3]. The mechanisms underlying drug-resistance in epileptic patients are still unclear; inter-individual differences in receptor expression patterns or receptor sensitivity to anti-seizure drugs have been proposed as possible explanations. An additional hypothesis contemplates the alteration of the blood–brain barrier preventing anti-seizure drugs from properly reaching their targets in the central nervous system [4]. While the pathophysiology of drug resistance remains far from being fully elucidated, refractory epilepsy lowers quality of life and increases psychosocial, neurologic, and mortality risks, which ultimately raise the cost of healthcare. Resective surgery, neurostimulation using deep-brain stimulation (DBS), vagus nerve stimulation (VNS), and responsive neurostimulation surgery (RSN) in drug-resistant patients are currently considered treatment options that may result in effective seizure management [5,6,7,8,9]. Yet, a significant portion of individuals are ineligible for resective surgery because their seizures are widespread or multifocal in origin or originate from eloquent or deep-seated brain regions. On the other hand, patients receiving non-resective neuromodulatory therapy seldom experience seizure-free periods and frequently need to take additional medications to keep their seizures under control [10].

There are common epilepsy syndromes that make patients suitable candidates for resective and non-resective interventions, such as mesial temporal sclerosis (MTS), focal cortical dysplasia (FCD), benign or low-grade tumors, cavernomas, tuberous sclerosis, and areas of encephalomalacia/gliosis [11]. MTS epilepsy associated with hippocampal sclerosis is the most common focal drug-resistant epilepsy syndrome [12]. This usually carries a highly suggestive history, characterized by atypical febrile convulsions, perinatal infections, or injury, followed by a long silent period with development of drug-resistant epilepsy later in adult ages [13]. The semiology of the seizures typically includes an aura consisting of epigastric sensation, followed by behavioral arrest with concurrent oroalimentary and upper limp automatisms [14]. The most common pathologic substrate of MTS is hippocampal sclerosis (HS), which is characterized by loss of pyramidal cells and severe astrogliosis, macroscopically corresponding to a varying degree of atrophy [15]. Such histopathological findings are paralleled, on MRI, by reduced hippocampal volume, local T2-weighted hyperintensity, and alteration of hippocampal internal structure [16].

Despite representing the most surgically treated epileptic syndrome, outcomes of surgical resections in MTS patients with HS are quite variable, supporting the hypothesis that such a condition is more complex and likely encompasses a spectrum of phenotypes. This concept has been supported by neuropathologic investigations revealing different patterns of neuronal loss within hippocampal subfields and in surrounding structures belonging to the mesial temporal lobe [9,17]. In particular, the ILAE task force identified three types of hippocampal sclerosis by measuring the degree of neuronal loss across hippocampal subfields [18,19]. Briefly, in HS type 1, which constitutes the most frequently encountered neuropathological phenotype, the CA1 segment is the most severely affected, with significant neuronal losses also in CA2, CA3, and dentate gyrus. In HS type 2, neuronal cell loss and gliosis mainly affect CA1, relatively sparing other hippocampal subregions. In HS type 3, CA4 and dentate gyrus are the most affected hippocampal regions, with only subtle pathological changes in other zones [9]. While patients affected by HS type 1 seem to develop early seizure onset and more favorable outcomes after surgery, patients with HS types 2 and 3 show differences in epilepsy onset and less satisfactory outcomes [20]. The surgical approaches in cohorts affected by mesial temporal epilepsy are quite heterogeneous (anterior temporal lobectomy, amygdalo-hippocampectomy) and the available evidence does not support a clear superiority of one approach when looking at the trade-off between seizure freedom and minor cognitive abnormalities as outcome measures [21]. However, a study conducted on a large number of patients recently proposed anterior temporal lobectomy as a better surgical approach; in this scenario, the authors proposed that a more extensive surgical approach would more easily influence distant nodes of an abnormal brain network, leading to better clinical outcomes [22].

Another commonly encountered indication for resective treatment is FCD, which is the result of a cortical maldevelopment leading to altered lamination, with or without cytoarchitectural abnormalities [23]. FCD may be located anywhere across the cortical mantle and account for drug-resistant epilepsy, the seizure semiology of which mainly depends on the location of the FCD itself. FCD-related epilepsy usually begins during early childhood, and it is characterized by high seizure frequency and marked drug resistance [24]. MRI features of FCD consist of increased cortical thickness, localized brain atrophy, blurring of the interface between gray matter and white matter at cortical level, and T2-weighted hyperintensities that can be radially detected (i.e., the transmantle sign) [25]. A new classification of FCD has been recently provided by the International League Against Epilepsy (ILAE) which separated isolated FCD types I and II from FCD with concomitant epileptogenic lesions (type III) [19]. The available evidence on the outcomes of FCD surgery suggests inferiority, to a certain degree, when compared to other etiologies usually treated with resective surgery. However, the result of this therapeutic approach seems to heavily depend on variables such as the precocity of the intervention, the histological subtype of FCD, and its location [23].

An ulterior target for resective treatment in the setting of drug-resistant epilepsy is represented by ganglioneuronal or low-grade glial tumors [26,27]. Indeed, gangliogliomas (GG) and dysembrioplastic neuroepithelial tumors (DNET) are increasingly recognized as a cause of epilepsy in young patients and may coexist with FCD, due to their maldevelopmental origin [26]. These tumors may be located throughout the brain, with the highest frequency in the temporal lobe; they usually have a biologically benign behavior, but sometimes malignant transformation has been reported. Neurosurgical series investigated outcomes and the prognostic factors of seizure control in patients affected by ganglioneuronal tumors. Despite the high heterogeneity across studies, especially regarding surgical procedures, a high rate of seizure freedom was observed after surgery, with higher percentages in patients who underwent early surgical treatment (<1 year of seizures), exhibiting only focal seizures without secondary generalization and when gross total resection was performed together with hippocampectomy and corticectomy [27]. Similar results have also been reported in patients with low-grade gliomas [28]. Among lesions that are the result of an erroneous developmental process, cerebral cavernous malformations (CCMs) constitute a frequent cause of focal, pharmacoresistant epilepsy. CCMs are congenital vascular lesions that can arise in any site of the central nervous system [29]. They may be isolated or multiple, sometimes associated with FCD and/or hippocampal sclerosis, thus configuring examples of dual/triple pathology. Epileptic seizures are the most common clinical manifestations in patients with CCMs, especially when they are located in supratentorial regions, adjacent to the cerebral cortex [30]. Specifically, epilepsy related to CCMs occurs when clinical, electroencephalographic and neuroradiological data converge on a CCM as the cause of epileptic seizures [30]. Although surgical treatment of a CCM is recommended as early as possible, even if the ILAE criteria for drug resistance are not strictly satisfied, seizure freedom after surgery is reached in only in 75% of patients. Such a relatively low rate may be explained by heterogeneity of cavernoma localization, the variety of surgical procedures currently applied, and a history of cavernoma-related epilepsy [29,30].

Finally, the tuberous sclerosis complex (TSC) represents a rare neurocutaneous syndrome that is characterized by the presence of tubers that serve as epileptogenic foci [31]. As with other conditions, resective or palliative surgery is indicated when two trials of anti-seizure drugs are unable to control epileptic seizures [32]. Although a specific antiepileptic drug exists, data on surgical series suggest that up to 55–60% of patients with TSC who underwent surgery achieved seizure freedom. Surgical treatment of TSC is made particularly complex by the presence of multiple tubers and the subsequent need to select the most “epileptogenic” one, with the persistent uncertainty of removing the surrounding cortex. These issues may be solved by employing advanced neuroimaging techniques that are currently unavailable in common clinical practice in most centers [31,32].

Taken together, the available data on resective surgery applied to different epilepsy etiologies suggest that:

(i) surgical treatment is safe and helps to achieve seizure freedom in a non-negligible percentage of patients;
(ii) notable differences exist in terms of outcomes across different etiologies; and
(iii) variables such as lesion location (e.g., temporal vs, extratemporal), duration of epilepsy before intervention, and seizure semiology (e.g., strictly focal vs. focal to generalized) influence the results of resective therapy within each etiological subgroup.

Starting from these premises, it is worth noting that a resective approach cannot be applied to a conspicuous number of patients affected by drug-resistant epilepsy [33]. Indeed, a clearly delineated epileptogenic zone is not always identified by using the available neuroimaging techniques. Sometimes, even if identified, the putative cause of drug-resistant epilepsy is located within eloquent brain structures, where performing a radical surgery may be harmful to normal brain functioning. This is especially true for individuals carrying combined pathology (e.g., HS and FCD), necessitating a more complete investigation to determine the size of the epileptogenic zone or the lesions that are “more epileptogenic than others”. Given that epilepsy, like many other neurological disorders, is currently modeled as a network disorder, some previously mentioned neurostimulation techniques (VNS, DBS, and RNS), are thought to act both on specific circuits and on altered networks at the system level, and are currently gaining credit as an alternative in drug-resistant patients, further widening the horizon of the alternatives available to decrease seizure frequency and quality of life in this group of patients. Finally, prior to prospecting any surgical intervention for patients affected by drug-resistant epilepsy, the clinician must ascertain the effective presence of a drug resistance. Indeed, seizures may continue to occur if the pharmacological therapy is not properly administered by the patient or if the selected drug is not well-suited to treat the specific epileptic syndrome at hand. Thus, compliance with therapy and the choice of the anti-seizure drug need to be carefully reviewed [34]. In addition, psychogenic non-epileptic seizures (PNEs) need to be ruled out before definitively labeling epilepsy as refractory to medications [35].

## 2. Conventional Non-Resective Interventions for Intractable Diseases

Over the last 40 years, three non-resective neuromodulatory procedures have been developed for patients with medically intractable epilepsy and epilepsy syndromes with underlying lesions for improving seizure control [7,33].

(a) Vagus nerve stimulation (VNS): During the 1990s, when VNS first became available, it produced good outcomes, together with the longest availability period. It is a less invasive technique, where a generator is inserted subcutaneously, typically in the left chest region, and an electrode is inserted with the electrode’s end wrapped around the contralateral vagus nerve. The generator’s stimulation is intermittently timed by an experienced physician. To optimize specific devices, a wand device attached to a hand-held computer system can change the voltage and timing of the stimulation. According to a large series, between 30% and 50% of patients who received VNS had a successful outcome, with a seizure reduction of more than 50% [36]. The stimulation might cause headaches, coughing fits, and voice alterations. By altering the stimulation intensity parameters, these can be reduced.
(b) Deep-brain stimulation (DBS): Throughout time, DBS has proven to be successful in treating movement disorders. The ventralis intermedius nucleus of the thalamus, the subthalamic nucleus, and the globus pallidus pars interna are frequently stimulated in these illnesses [37]. The ipsilateral mesial temporal lobe and the hippocampus, but not the lateral temporal lobe or the contralateral hemisphere, are directly impacted by DBS stimulation of the anterior thalamus. According to a study by Zumsteg et al. [38], with a rate of seizure reduction of 20 to 92%, earlier trials demonstrated this treatment’s effectiveness in treating medically refractory epilepsy [39]. Most recently, the Stimulation of the Anterior Nucleus of the Thalamus in Epilepsy (SANTE) study was completed across multiple centers and was double-blind, randomized, and multicentered [40]. While considering DBS surgery for the treatment of epilepsy, it is important to consider the surgery’s tiny but significant risks, such as focal bleeding at the probe implantation site.
(c) Responsive neurostimulation system (RNS), In the past 20 years, the United States has developed a method for directly detecting seizures in the skull and stimulating the brain directly [41]. In 2013, the US Food and Drug Administration granted approval for an implantable brain-responsive neurostimulator known as the RNS system (Mountain View Inc., CA, USA). The RNS system is an adjuvant therapy for people with focal onset seizures that are medically uncontrolled and limited to one or two epileptogenic foci. This technology is the first-ever closed-loop epilepsy neuromodulation system [42]. It continuously checks for epileptiform activity at or less than 2 cm from seizure foci and responds with electrical stimulation when it is found.

All these neuromodulatory interventions offer an alternative, palliative approach to the treatment of medically refractory epilepsy. However, patients are rarely seizure-free and often still require medications to maintain control of their seizures after these non-resective treatments [7].

## 3. The Application of MRgFUS in Epileptic Patients

With the advancement of technology and the ongoing search for better, less invasive neurosurgical techniques, magnetic-resonance-guided focused ultrasound (MRgFUS) has been proposed as an efficient and minimally invasive therapeutic ablation of tissue to disconnect aberrant networks and/or as an adjuvant therapy to modify brain networks in neurological patients [43,44]. Radiofrequency ablation and, mainly, MRI-guided laser interstitial thermal therapy (LITT) are minimally invasive alternative surgical options to MRgFUS treatment. Using heat produced by light absorption, LITT is a technique for tissue destruction. A stereotactic approach is used to implant a tiny diameter fiber optic applicator into the lesion in order to provide this energy in a minimally invasive manner. Using a catheter that is cooled by water or saline, light energy is passed into the lesion region and, then, converted to heat by the tissue. Although still requiring skull and brain penetration, ablating brain tissue in real time with LITT is becoming more acceptable for epileptic patients as a well-considered and effectively tested option [45].

Historically, the first controlled, well-defined, and targeted ablations in mammalian cortical and subcortical regions were carried out by Lynn and Putnam in the early 1940s [46]. Fry et al. [47] used high-intensity ultrasounds in the 1950s to cause focused necrotic lesions in the brain basal ganglia of previously craniotomized animals without damaging the surrounding brain parenchyma. In order to deliver targeted ultrasound straight into the brain parenchyma at this earlier stage, a craniotomy was unavoidable. It has been possible to transfer sufficient thermal energy in a precise focus through the intact skull and straight to deep-brain areas to cause a controlled thermal ablation, thanks to the invention of devices made up of several phased-array transducers [48]. The development of MRI proton resonance frequency shift thermometry, which is used to track the release of energy in the targeted region and surrounding tissues, as well as the ability to precisely control the delivery of acoustic energy, has improved the accuracy and safety of MRgFUS brain ablation and paved the way for so-called non-invasive functional neurosurgery [49]. 

Since the 1990s, there have been a number of useful applications of minimally invasive techniques like FUS, including high intensity focused ultrasound for ablation [50], peripheral nerve blocking [51], and stroke thrombolysis [52]. For the development of neurotherapeutics, the targeted disruption of the blood–brain barrier to enhance drug delivery also represents a very intriguing advantage, using low-frequency sonication [53]. As a cutting-edge method of brain stimulation, low-intensity focused ultrasound (LIFU) has a great deal of potential. The effects of even brief sonication may endure for many hours [54] and, unlike transcranial magnetic and electrical stimulation, LIFU can directly influence activity inside deep-brain areas with high spatial precision [55], using lower frequency (about 220 kHz) to modify neuronal activity in a reversible manner [7,56]. Ultrasound can interact with tissue to cause mechanical and thermal consequences. According to one theory, the mechanosensitive ion channels and voltage-gated calcium, sodium, and potassium channels in neuronal membranes become more permeable when exposed to modest levels of acoustic waves force [57]. Another theory holds that mechanical changes in the tension of the plasma membrane or the lipid bilayer caused by vibration of the extracellular and intracellular surroundings regulate the activity of neurons [58]. The temperature increase caused by LIFU is frequently 0.1 °C; therefore, the effects are probably insignificant, even if an increase in tissue temperature could impact neuronal activity (this is the mechanism of action in irreversible ablative high-intensity focused ultrasound).

The Food and Drug Administration (FDA) has previously approved the use of a high-intensity focused ultrasound (HIFU) system operating at high frequency (650 kHz) as a surgical treatment for a number of illnesses, including bone metastases, adenomyosis, and uterine fibroids. In the neurological realm, MRgFUS has received FDA and European Commission approval for essential tremor, Parkinson’s disease, and neuropathic pain, respectively [59,60]. The use of FUS over an intact skull and advances in magnetic resonance imaging thermometry make MRgFUS an appealing therapeutic approach that is being investigated for a variety of additional neuropsychiatric diseases, neuro-oncological pathologies, and neurological illnesses, including epilepsy. In the treatment of epilepsy, MRgFUS represents a non-invasive transcranial procedure that has several potential roles: ablative procedures to damage the epileptogenic zone and/or disconnect the epileptogenic networks using HIFU and neuromodulation of brain networks or disruption of the blood–brain barrier (BBB) using LI-FU [7,61,62]. 

During a MRgFUS treatment, the patient must be awake and cooperative in order to receive prompt feedback on his or her health and complete a neurological examination at the conclusion of each sonication. Although 3T MRI scanners were initially used for MRgFUS procedures, it has now been shown that they can also be safely and effectively used in 1.5T MRI scanners [63], which allows for high-resolution intraoperative imaging, because a dedicated MR coil is not yet available for 3T integrated MRgFUS systems [64].

### 3.1. Role of MRgFUS in Epileptic Patients: High-Frequency Ablative MRgFUS

The aberrant epileptogenic zone excised in conventional epilepsy surgery is wider and typically necessitates a cortical resection or a lobectomy, in contrast to the small surgical area that is traditionally targeted in movement disorders using standardized procedures. Every minimally invasive or noninvasive method that eliminates the epileptogenic focal dysfunction without requiring a craniotomy or brain penetration is particularly appealing, because the traditional surgery is substantial and not exempt from complications. For patients with mild temporal lobe epilepsy (MTLE), in the most frequent type of epilepsy surgery the amygdala and hippocampus are routinely removed, along with or without excision of the temporal neocortex. 

MRgFUS could become an alternative method to target the area of mesial temporal (epileptic focus) dysfunction and produce a functional outcome comparable to that of resection of mesial temporal structures (the amygdala and the hippocampus). However, the anatomical intricacy of the medial temporal structures should be taken into consideration before considering MRgFUS to treat MTLE. Indeed, it is difficult to effectively ablate the target while protecting the delicate skull-base structures, significant blood arteries in the sylvian fissure, the cranial nerves, and the brainstem [65]. Using three cadaveric skulls, Monteith et al. [65] sought to evaluate whether lesional temperatures could be reached in the target tissue of the mesial temporal lobe, evaluating any potential safety issues with the MRgFUS operation. Generally, the target regions (including the amygdala, the uncus and the nearby parahippocampal gyrus) represented an ablative volume of 5 cm^3^. To track the temperature of the important skull-base components, they used thermocouples, discovering that lengthier sonications (30 s) were necessary to reach temperatures up to 60.5°, which would completely obliterate the targeted brain tissue. Regrettably, the lengthier sonications resulted in heating of the skull-base structure (up to 24.7°). In a recent study, Parker et al. [66] developed a noninvasive MRgFUS ablation strategy for treating patients with mesial temporal sclerosis. The idea was to disconnect posterior hippocampal tracts as an alternative strategy to amygdalohippocampectomy. For this reason, these authors reviewed 3T MRI DTI scans of two patients with mesial temporal sclerosis in order to target a different anatomical site with respect to the well-known anterior nucleus (AN), which represents the main MRgFUS target for non-resective interventions in refractory epilepsy. Within the limbic circuitry, there is the fornix–fimbria posterior pathway, a white-matter tract that has been discovered to have MTS-related axonal damage and diffusion anomalies [67]. They showed that traditional MRI DTI scans may be used to plan patient-specific target lesions, with a margin of safety from the ocular radiations, by modeling the fornix–fimbria pathway and the critical nearby structures using DTI, increasing accuracy and safety.

Regarding the ablative procedure, the first clinical application of MRgFUS on an epileptic patient was made by Abe et al. [68]. They showed promising evidence of the beneficial effect of MRgFUS for seizure control in a 36-year-old right-handed woman with complicated MTLE. Because selective hippocampectomy carries a higher risk of brain impairment, she was hesitant to have it peformed. In addition, she declined the recommendation of gamma-knife surgery, due to the possibility of cerebral edema (a frequently documented adverse consequence). For this reason, the patient accepted a left-sided hippocampal MRgFUS to treat her MTLE. MRgFUS ablation was performed over the left hippocampus (the target was situated 15 mm above the base of the skull and 20 mm laterally from the midline). At a 1-year follow-up, the patient had improved her quality of life and was seizure-free after the treatment [68].

Treatment of epilepsy linked to minor or subcortical targets, such as cortical malformations or hypothalamic hamartomas, might be another rationale for MRgFUS. A 26-year-old man with epilepsy brought on by a sessile para hypothalamic hamartoma underwent a successful MRgFUS ablation as part of a disconnection procedure in 2020, according to Yamaguchi et al. [69]. At the 1-year follow-up period, the patient was seizure-free while taking fewer antiepileptic medications. Contrary to stereotactic radiosurgery, however, it is far more challenging to anticipate the extent of a lesion and the perilesional edema it causes. 

### 3.2. Role of MRgFUS in Epileptic Patients: Low-Frequency Sonication/Neuromodulation

One of the potential benefits of MRgFUS is its capacity to control brain functioning without actually creating a lesion. In comparison to neurostimulation with DBS, MRgFUS has the advantage of not requiring gear, like depth electrodes and pulse generators. This is because it uses low-frequency sonication. If additional therapy is required, it can likely be performed safely. Focused ultrasound has been found, in studies, to be effective at blocking nerve conduction [51,70] and may be used to treat neurological illnesses involving diseased networks. This method would have the ability to disrupt or modify these networks, as epilepsy is typically regarded as a network disorder. In the future, the definition of connectome profiles in epileptic brains could aid in defining the total seizure genesis and propagation as a complex overlay of cortical–subcortical network interactions [71]. 

Low-frequency ultrasound MRgFUS is currently being investigated in human trials and animal studies, since it can facilitate chemotherapy, stem cell, or gene therapy delivery to focal brain targets by disrupting the BBB through expansion of microbubbles [71]. This suggests a future option for MRgFUS-mediated delivery of biological agents, in addition to its applications for epilepsy. The distribution of numerous antiepileptic medicines could be made easier and more effective because of the promising ability of ultrasound-induced BBB disruption. Patients with refractory autoimmune encephalitis, autoimmune epilepsy, or newly diagnosed refractory status epilepticus might benefit from the delivery of medicines by ultrasonography [72].

Low-frequency sonication offers several advantages over high-frequency sonication, such as the potential to cause non-destructive, reversible neurophysiological alterations. Without requiring a surgical procedure or the insertion of a device, ultrasonic energy could function similarly to a treatment involving deep-brain stimulation or neurostimulation. As stated by Kamimura et al. [73], FUS may induce a deformation of the neuronal membrane that can mechanically couple with the endogenous mechanical waves linked to action potentials, thereby disrupting membrane electrical depolarization. Alternatively, it has been proposed that the cellular temperature variations induced by FUS may affect synaptic potentials and neuronal membrane conductance. The idea behind this approach is that we could be able to remodulate abnormal reorganizations of neural networks and reduce epileptic bursts. In order to evaluate the ultrasound neuromodulation mechanisms determined by low-frequency FUS in epileptic brains, Zhang et al. [74] applied this treatment in the hippocampus of epileptic rats, in which seizures were induced by kainic acid. They found that the functional brain network was dramatically altered by low-frequency FUS, particularly in the low-frequency range. After sonication, the strength of the connections between various brain regions considerably diminished when compared to the connection strength in the control group. The authors hypothesized that low-frequency ultrasound suppressed epileptic signal transmission by altering the connections in the theta-band brain network, which in turn prevented epileptic seizures. Thus, macro- and meso-scale circuit dynamics may be affected by ultrasound by altering brain functional connections, particularly using acoustic frequencies ranging between 0.25 and 0.65 MHz, which serve as the hippocampus’ primary frequency range. This finding was also confirmed by Hakimova et al. [75], demonstrating that epileptic mice who underwent this kind of treatment targeting hippocampal neural activity showed fewer spontaneous recurrent seizures.

Another specific target for applying low-frequency FUS in epileptic patients is the thalamus. The role of the thalamus in pathophysiological mechanisms of epilepsy—both generalized and focal epilepsies—is well-known [76]. The lateroventral anterior nucleus (AN) of the thalamus, which is adjacent to the mammillothalamic tract, was reported to be the most effective in controlling seizures [76]. Using DBS, Krishna et al. [77] found that the best method for stopping seizures was to stimulate the lateroventral AN close to the mammillothalamic tract. The first translation of this DBS-related evidence to a FUS neuromodulation approach was proposed by Min et al. [78], who found that transcranial low-frequency sonication of the thalamus reduced epileptic burst in an animal study in which epileptic seizures were chemically induced acute epilepsy.

## 4. Conclusions and Future Directions

While the main clinical complications following MRgFUS in epileptic patients are still unknown, the information presented in this work indicates that ultrasound-based technologies have significant clinical prospects. On one hand, MRgFUS may, at least theoretically, become a valid alternative to invasive surgical strategies such as resection or neuromodulation via DBS. Indeed, it carries the advantage of creating lesions with notable spatial precision under controlled conditions, without requiring craniotomy or direct access to brain parenchyma. Despite these premises that make MRgFUS lesional therapy for refractory epilepsy captivating, its clinical relevance is currently constrained by a number of factors. As discussed earlier in the paper, MTLE, which constitutes the major referral for surgical treatment, underlies a large epileptogenic focus and surrounding zone (volume = 5 cm^3^), requiring quite substantial removal of brain parenchyma, which may be barely achieved by MRgFUS without potentially damaging skull-base or neighboring structures. Even if lesions induced by MRgFUS may not encompass large brain areas with the currently available technology, a possible alternative is targeting pathophysiologically meaningful small nuclei or white-matter tracts, in a way that is similar to strategies that are currently tested in the movement-disorder field. Targeting the thalamic AN is a currently studied target for DBS in drug-resistant epilepsy [79]. In addition, inducing focal lesions to fornix–fimbria and posterior hippocampal tracts, resembling tractotomy of pallidothalamic tracts, may be another viable strategy. Nowadays, there are only anecdotal descriptions of such approaches in sporadic case reports. Indeed, it requires a detailed knowledge of seizure origin and spreading patterns to fully implement such strategies in well-designed, controlled, clinical trials. For these reasons, more preclinical studies are advocated to identify which targets may constitute suitable candidates for ablation. Such steps will require the usage of advanced MRI techniques to allow reliable identification of structures that are not clearly distinguishable, using commonly available MRI sequences together with potential MRI biomarkers. However, a large amount of multicenter clinical trials are under way (NCT03417297, https://clinicaltrials.gov/ct2/show/NCT03417297, accessed on 1 October 2023 and NCT05032105; NCT 02151175, https://clinicaltrials.gov/ct2/show/NCT02151175, accessed on 1 October 2023; https://clinicaltrials.gov/ct2/show/NCT05032105, accessed on 1 October 2023; NCT02804230, https://clinicaltrials.gov/ct2/show/NCT02804230, accessed on 1 October 2023). These studies will open new perspectives for determining the feasibility, safety, and efficacy of MRgFUS ablation of epileptic foci. 

On the other hand, low-intensity FUS neuromodulation may be a valid alternative to more widespread neuromodulation techniques such as TMS and tDCS. Despite having temporary effects, delivery of focused ultrasound to deep-brain structures may provide insights into seizure origin and propagation. Indeed, temporary disruption of abnormal activity spreading across brain networks may potentially inform invasive treatment strategies as well as lesion-based interventions. Moreover, low-intensity FUS may have a therapeutic role itself, by aborting the emergence of seizures. However, more research is needed to understand the biochemical mechanisms behind seizure control. It is possible that the mechanical forces brought on by FUS may disrupt the synaptic contacts and stop epileptic discharges from spreading. Preclinical studies are needed to shed light on these mechanisms. Future directions are closely related to technological advances. Among these, histotripsy may represent a new frontier of non-resective treatment, as suggested by Krishna and colleagues [62]. The term histotripsy was first used in 2004 [80] and is a noninvasive focused ultrasound technology that uses brief ultrasound bursts (microseconds in length) with a low duty cycle (1%) to minimize heating and higher peak pressure amplitudes. Such a technique induces microablations, generating acoustic cavitation from endogenous gas in tissues [62]. 

In conclusion, the landscape of focus ultrasound techniques’ possible applications is currently widening. Despite being an “old” technique, it has recently gained attention due to technological advances allowing it to deliver ultrasound in a safe and effective way. Its intrinsically non-invasive nature makes it a fascinating treatment opportunity, along with resective and non-resective strategies in patients affected by drug-refractory epilepsy. Despite being at its dawn, advances in the preclinical field, as well as neuroimaging and neurophysiology techniques, make its growth likely to be considerable in the future years, leading to novel ways to improve seizure control in such a challenging group of patients.

## Data Availability

Data are contained within the article.
